# Nanocarriers in Ungual Drug Delivery

**DOI:** 10.3390/pharmaceutics17081060

**Published:** 2025-08-15

**Authors:** Sheila Porto de Matos, Karen de Oliveira Araujo, Tainá Kreutz, Valdir Florêncio da Veiga Júnior, Helder Ferreira Teixeira, Letícia Scherer Koester

**Affiliations:** 1 Programa de Pós-Graduação em Ciências Farmacêuticas, Faculdade de Farmácia, Universidade Federal do Rio Grande do Sul, Av. Ipiranga, 2752, Porto Alegre 90610-000, Brazil; 2 Programa de Pós-Graduação em Química, Instituto Militar de Engenharia, Praça General Tibúrcio, Urca, 80, Rio de Janeiro 22290-270, Brazil

**Keywords:** ungual drug delivery, nail disorders, nanostructured systems, onychomycosis, psoriasis

## Abstract

Ungual disorders can impact quality of life, with onychomycosis and nail psoriasis being the most prevalent disorders among the general population. In humans, the main functions of the nail apparatus comprise protection against trauma, improvement of tactile sensations, and allowing precision gripping. In order to perform such functions, the nail plate has a hard structure formed by dead keratinized corneocytes tightly bound to each other, giving the nail plate a “barrier-like” character. Due to this property of the nail plate, drug delivery to the region is hindered, making the treatment of ungual disorders difficult, either by systemic or topical drug administration. Many strategies have been developed in the last few decades in an attempt to increase the bioavailability of drugs in the nail. Interest in the employment of nanostructured drug delivery systems aiming to increase the bioavailability of drugs in the nail plate upon topical administration has increased. Moreover, the association of the nanotechnological approaches with other methods may be a beneficial strategy when aiming to increase drug permeation through the nail barrier. In this sense, the present review has the intention of presenting the panorama of the current technological development of nanostructured systems designed for the local treatment of ungual disorders. Through this extensive literature review, it was possible to recognize, among the studies, a lack of standardization regarding the methodology of nail permeation assessment, which imposes an obstacle to comparison.

## 1. Introduction

Ungual disorders can range from harmless morphological modifications to painful and chronic symptoms [1,2,3]. The most prevalent ungual diseases are onychomycosis (fungal infections mostly caused by dermatophytes) and nail psoriasis (an ungual manifestation of psoriasis, its usual symptoms are nail pitting and onycholysis) [4,5]. Structurally, a nail unit comprises the nail plate, nail folds, nail matrix, nail bed, and hyponychium [1,6,7].

The nail plate is formed by the nail matrix and grows in the direction of the hyponychium, where it detaches from the nail bed [1,3,8]. The nail plate, the most visible part of the nail unit, is a hard structure formed by dead cornified cells and divided into dorsal, intermediate, and ventral layers, which represent a significant barrier, imposing challenges to the drug delivery in the region [1,6,8,9,10].

Drug delivery, by topical or systemic routes, faces challenges due to the difficulty of permeating through the nail plate and reaching therapeutic concentrations at the site of infection [3,11]. Several strategies are currently employed to enhance drug permeation through the nail barrier. Numerous methods are described in the literature, including mechanical, physical, and chemical techniques [1,4,10,12]. Notably, recent investigations have shown that nanostructured systems can be used for ungual drug delivery. These systems are already widely employed in transdermal drug delivery research with promising results and can be potential candidates to enhance the transungual permeation of bioactive substances [3,13,14]. Nanocarriers can be associated with physical permeation-enhancing techniques [15,16,17,18] and/or contain permeation enhancers (PE) in the formulation [19,20,21,22,23,24,25,26,27,28,29] to achieve even more positive results.

Nanostructured systems stand out as valuable tools for enhancing drug delivery and the stability of drugs, often presenting better performance in terms of efficacy for ungual administration compared to “free” drug administration. The increase in contact area, the presence of permeation-enhancing excipients, and the ability to modulate surface charge can favor permeation through the nail plate [30]. Additionally, these systems enable higher drug retention and controlled release at the target site, reducing the need for high doses, repeated/recurrent administration, and minimizing the risk of side effects compared to systemic administration [31,32]. Unlike permeation enhancement techniques such as mechanical and physical, which may be invasive and potentially damage the nail structure, nanocarriers act primarily through physicochemical interactions with the nail plate, preserving its structural integrity [33].

Currently, there is no review in the literature focusing on the research concerning the development of nanostructured systems as a tool in achieving drug delivery to the nail. Thus, this work discusses the current status of research concerning the use of nanotechnological approaches in the design of pharmaceutical dosage forms for ungual drug delivery and also highlights the importance and potential impact of the incorporation of nanostructured systems in vehicles for ungual administration.

## 2. Nail Anatomy

**Figure 1:**
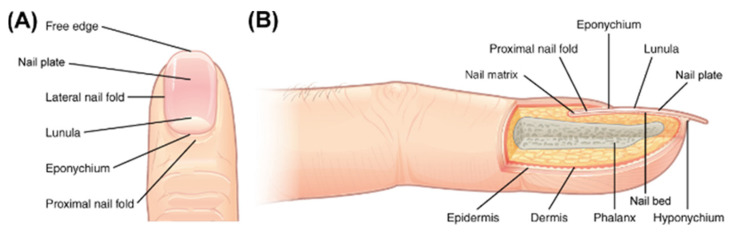
Top (**A**) and lateral (**B**) views of the nail apparatus. Adapted from OpenStax College CC BY 3.0 (https://creativecommons.org/licenses/by/3.0).

The human nail is a structure positioned in the distal portion of fingers and toes, and its main functions are protection from potential trauma, improvement of tactile sensation, and allowing precision gripping [10,34,35]. The nail unit (Figure 1) is divided into the nail plate, nail matrix, nail bed, nail folds, and hyponychium. In the most proximal part of the nail unit is localized the nail matrix, made of germinative epithelium from which the nail plate is generated. The nail plate grows distally, with the nail bed, a thin vascularized epithelium, underneath it. The nail plate is held laterally and proximally by the nail folds. Finally, the region where the nail plate detaches from the nail bed is the hyponychium [1,3,8,9]. The nail plate, the most visible part, is a translucent, hard but malleable curved structure with a thickness varying from 0.25 mm to 0.6 mm, and is produced by the maturation and keratinization of the nail matrix epithelium. During keratinization, the cells undergo changes similar to those of epidermal cells that build the *stratum corneum* [36]. However, the nail plate is approximately 100 times thicker than the *stratum corneum* and is composed of keratin fibers embedded in an amorphous protein matrix [37]. Keratins are insoluble proteins comprising sulfur-rich polypeptide chains capable of forming filaments [38]. These chains may have a helical conformation (α-keratin) or a sheet-like structure (β-keratin). In mammals, α-keratins are the main components of hair, nails, hooves, horns, and the epidermal layer of the skin. They are subdivided into two groups: “hard” α-keratin (hair type) and “soft” α-keratin (skin type). Hard α-keratin has a higher cysteine content with numerous cross-linked chemical bonds between the formed filaments and the amorphous matrix, making it more stable than soft α-keratin [8,11].

The nail plate is composed of keratin fibers. Keratin fibers are organized into three layers: dorsal, intermediate, and ventral. Their relative thickness ratio is 3:5:2, respectively [39]. This sandwich-like orientation provides the nail plate with hardness and rigidity. The dorsal and ventral layers contain soft keratin filaments oriented parallel and perpendicular to the direction of nail growth. The intermediate layer contains hard keratin filaments oriented parallel to the nail plate surface but perpendicular to the growth axis [8]. Nail growth rate is affected by various factors, including age, gender, nutrition, and trauma. On average, fingernails grow at a rate of 0.1 mm/day, while toenails grow at a rate of 0.03–0.05 mm/day, taking about 6 months and 12 to 18 months, respectively, for complete regrowth [40].

The nail plate also contains significant amounts of water, ranging from 14 to 30% (*w*/*w*), depending on relative humidity. Hydration is an important factor in maintaining the nail’s elasticity and flexibility [41]. As a result, the nail plate is often described as a hydrophilic gel membrane [39,42]. The lipid content, found mainly in the dorsal and ventral layers, ranges from 0.1 to 1% and includes cholesterol sulfate, ceramides, free sterols, free fatty acids, triglycerides, sterol esters, and squalene [43]. The keratinized and rigid composition of the nail hinders drug delivery and bioavailability at the affected site [44]. This structure can be divided into three layers:

1. The dorsal layer, a poorly permeable surface, comprising overlapping cells. This layer is just a few cells thick [45].
2. The intermediate layer, the thickest layer, is softer and more malleable.
3. The ventral layer, a thin layer whose function is to connect the nail plate to the nail bed underneath.

The dorsal and intermediate layers are produced by the nail matrix, whilst the ventral layer is produced by the nail bed [1,3,7,34,46]. Chemically, proteins are the major component of the nail plate, especially low-sulphur keratin filaments and high glycine/tyrosine proteins that form a polymeric matrix, with a water content of approximately 10% varying with relative humidity, and low amounts of lipid. This gives the nail plate a hydrogel-like behavior [1,7,8]. Due to its structural and chemical properties, the nail plate behaves as a barrier that prevents the permeation of substances across its extension and towards the nail bed, with consequent challenges to the delivery of drugs in this region upon topical administration of formulations [1,3,7,12].

## 3. Ungual Disorders and Current Therapy

A variety of disorders can affect the nail apparatus, from inoffensive morphological modifications such as pigmentation, discoloration striation, to painful and disabling conditions. The two most common and clinically relevant nail diseases are onychomycosis and nail psoriasis [1,2,3,4,5].

Onychomycosis is a fungal infection of the nail apparatus mainly caused by dermatophytes and accounts for approximately 50% of nail disorders. It is prevalent in 40% of the world population and is increasing. This tendency is addressed by some authors as a reflection of the growth in elder, diabetic, and immunosuppressed populations, which are more susceptible to the condition, and by other social factors that increase the risk of nail fungal infection, such as use of common recreational facilities, sport activities, clothing choices, and hygiene habits [1,6,7,47,48,49].

The current treatment of onychomycosis involves systemic and topically administered drugs, used alone or in combination, depending on the severity of infection. Systemic oral therapy usually has low cure rates and high relapse rates of infection due to the region’s limited blood supply. Hence, the drug has difficulty reaching the site of infection in therapeutic concentrations. Additionally, antifungal drugs, currently sold, can present adverse effects such as hepatotoxicity. Drug interactions and long treatments can take months to achieve results, which presents the challenge of low patient compliance. In contrast, topical therapy can potentially overcome the side effects and increase patient compliance with the treatment. However, it faces challenges owing to the barrier characteristics of the nail plate. This leads to poor drug permeation and consequently low drug concentrations in deeper layers of the nail. Hence, topical therapy is limited to superficial infections. To expand the use of topical antifungal therapy in onychomycosis, there is a need to develop new strategies to enhance drug permeation through the nail plate and achieve efficient concentration on the infection sites [1,3,4,11,47].

Psoriasis is an autoimmune disease that affects epidermal and dermal cells. Its manifestation in the nail apparatus, with symptoms like nail pitting, onycholysis, and nail discoloration, is called nail psoriasis. Nail psoriasis can have an impact on the social life of patients, since it can cause an aesthetic impact and, if left untreated, it can lead to functional impairment and hamper the performance of daily chores [5,50,51,52]. It is estimated that nail psoriasis will affect nearly half of patients with skin psoriasis and up to 90% of patients with psoriatic arthritis. Approximately 5% of patients will present isolated nail psoriasis, without skin or joint involvement [5,51]. Although a variety of options for the clinical management of psoriasis are available, there is still a lack of standardized protocols for the clinical management of nail psoriasis. The treatment will depend on the individual case of the patient, considering the involvement of the skin and joints and the severity of the symptoms. It is not uncommon to overlook the nail symptoms, since they are difficult to treat and skin and joint manifestations seem to receive more attention in clinical routines [1,5]. In cases of isolated nail psoriasis, treatments include topical treatment with corticosteroids, vitamin D3 analogues, fluorouracil, laser treatment, photochemotherapy, and, in cases of more severe lesions, the intralesional injection of steroids.

As previously noted, the topical administration of drugs intended for the topical treatment of nail psoriasis in general is prescribed for less severe cases and tends to require longer treatment periods. This implies that patient compliance with the treatment can be a concern. Systemic therapy is considered in patients unresponsive to topical, intralesional, and phototherapy since drugs used in this modality can present side effects and impact patient compliance with the treatment [5,50,51,53].

As aforementioned, the topical treatment of ungual disorders is impaired by intrinsic characteristics of the nail that lead to poor permeation of drugs and/or bioactive molecules across its structure, consequently making it difficult to achieve therapeutic concentrations on the diseased areas. In order to overcome this problem, many strategies are used in an attempt to increase permeation of drugs through the nail plate, including mechanical, physical, and chemical methods, which are finely presented in previously published reviews (Table 1) [1,3,11].

Finally, recent studies have employed formulation strategies as tools to achieve better permeation of molecules across the nail barrier, such as nanostructured systems associated or not associated with chemical permeation enhancers and physical or mechanical methods [3,10,55]. In this context, the following sections of this review will describe and critically discuss the current status of the development of nanostructured systems, focusing on the topical treatment of ungual disorders, combined with or not combined with other techniques used to enhance transungual drug delivery.

## 4. Literature Survey

To access the current status of research concerning nanotechnological approaches to drug delivery to the nail, a literature survey was carried out using three bibliographic databases, namely Embase, Scopus, and Web of Science, and the database Espacenet for patent search. All scientific papers and patents published prior to 31 December 2024 were considered. Aiming to optimize the search results, database query lines were constructed using terms related to ungual drug delivery/nail pathologies AND nanostructured systems in the title and/or abstract.

The overall number of results obtained across the three databases was 484: 172 from Embase, 275 from Scopus, and 37 from Web of Science. A screening of abstracts of the resulting papers was made to investigate the compliance with all the following selection criteria:

- Original research;
- Written in English;
- Nanostructured systems;
- Formulation designed for topical administration to the nail unit.

**Figure 2:**
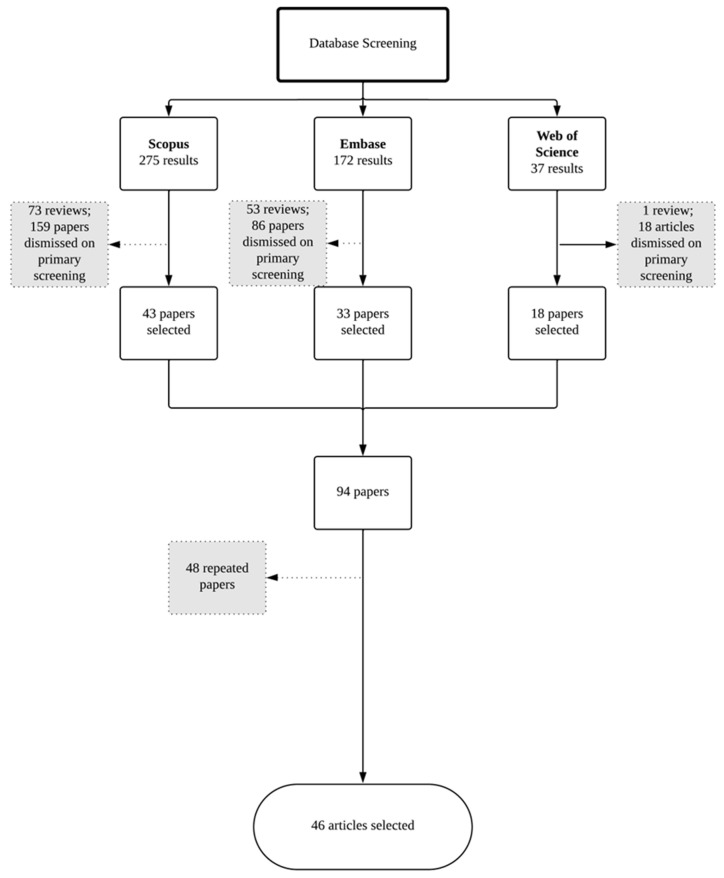
Flowchart demonstrating the steps of literature survey in scientific databases.

Query lines can be found in Supplementary Materials S1. All the papers complying with the selection criteria were considered, and duplicates were dismissed, leading to a total of 46 papers (Figure 2), which were selected for data extraction (Table 2).

The selection criteria applied for patents were the same as those previously described in the selection of research papers. The patents selected are listed and summarized in Table 3. It was noticed that at the time of writing, there are still relatively few patents concerning nanotechnological approaches towards the local treatment of ungual disorders, indicating that there is a potential for exploring the topic and the possibility of invention protection.

**Figure 3:**
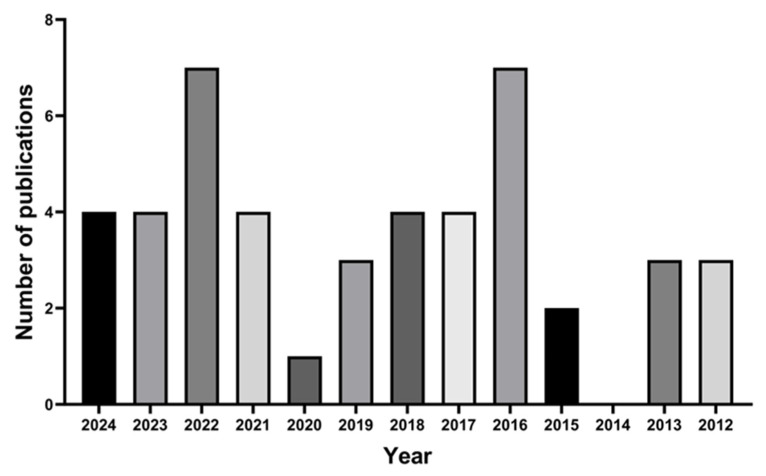
Annual distribution of research articles concerning nanostructured systems designed for ungual drug delivery.

Previously published reviews [1,10] have indicated that the interest in developing new therapeutic approaches to the local treatment of ungual diseases has emerged in the past few decades. The annual distribution of the selected papers (Figure 3) demonstrates that the development of nanostructured systems designed for ungual administration as a strategy to increase ungual drug delivery is recent and has increased during the last few years. The first report, published in 2012 by Barot, describes the development of a formulation containing terbinafine encapsulated in microemulsions and incorporated in a hydrogel designed for the topical administration to the nail in onychomycosis treatment [20].

**Figure 4:**
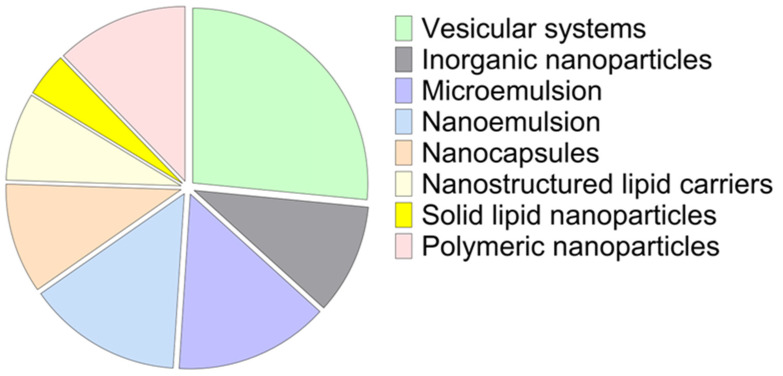
Nanostructured systems designed for the local treatment of ungual disorders.

In only two of the retrieved studies was no ungual disorder specified as a target [15,28]. All other studies focus on the treatment of onychomycosis. As shown in Figure 4, most selected studies address the ungual route of administration, with the transdermal (area adjacent to the nail) being the focus of a few studies.

## 5. Nanostructured Systems for Drug Delivery to the Nail

The concept of drug delivery through nanosized particles was first mentioned by the German Nobel laureate Paul Ehrlich at the very beginning of the 20th century, but the first reports of nanosystems developed for drug delivery were published only in the 1970s [86]. The first traceable report of nanostructured systems designed for topical administration dates from 1985 and is by Gurny et al. [87], who developed polymeric nanoparticles containing pilocarpine. From this time, the development of nanotechnology in the pharmaceutical field has grown gradually and currently represents an important field in pharmaceutical development. Searching bibliographic databases using terms related to “nanostructured systems” AND “topical drug delivery” will return thousands of hits. However, when limited to topical delivery to the nail, few papers are identified, and as displayed in Table 2, only in the last decade has the development of nanosystems for ungual drug delivery become an interest.

**Figure 5:**
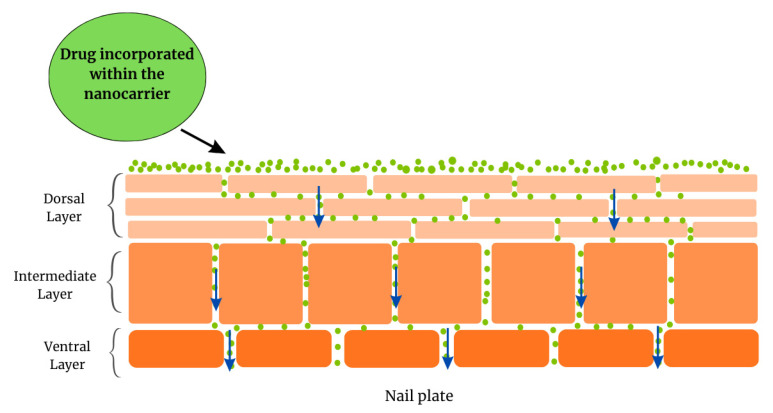
Schematic diagram of the nail plate illustrating a possible permeation pathway for a drug incorporated in a nanocarrier, where passage may occur through pores formed by multifactorial mechanisms.

Nanotechnology has proven to be a useful instrument in the field of therapeutics. Among the many possibilities, one can highlight abilities of nanostructured systems such as protecting the drugs against environmental factors that may cause degradation, enabling the administration of both hydrophilic and hydrophobic drugs, increasing drug bioavailability, providing controlled release of drugs, decreasing side-effects, and allowing improved permeation of drugs through biological barriers [31,88]. The use of nanosystems for transdermal drug delivery is already well established, widely reported, and well-reviewed concerning both technological aspects and the mechanisms involved in the permeation of the drugs through the skin barrier [31,32,89]. Nanostructured systems designed for local treatment of ungual disorders are presented in Figure 4 and discussed further in the following section. Detailed composition and particle size of example formulations are presented in Supplementary Materials S2–S8. In addition, Figure 5 presents a general diagram of the nail plate and a possible drug permeation mechanism.

### 5.1. Vesicular System [78]

VSs are nanostructured systems widely employed as drug delivery systems, including in drug delivery systems designed for topical administration. Liposomes are the most basic VS. New VSs have been developed in an attempt to enhance the permeation of drugs across biological barriers. VSs comprise lipid-based systems built from amphiphilic lipids organized in bilayers that surround an aqueous core. These are versatile since they allow the loading of both hydrophilic drugs (in the aqueous core) and hydrophobic drugs (within the lipid bilayer) [90,91]. VS classification encompasses a variety of systems, such as the following:

- Liposomes [90,92]: The simplest kind of VS, built fundamentally of phospholipids, cholesterol, and water [3,90,93];
- Transfersomes (TSs) and spanlastic vesicles (SVs): deformable and elastic VSs that, in addition to the LS components, contain surface active components that act by making the lipid bilayer more [3,90,93];
- Ethosomes (ESs): ESs contain ethanol in ranges of 20% to 45% in addition to the LS components. It provides flexibility to the particles and allows the entrapment of higher loads of drugs that might be inefficient in entrapment in other VSs [10,94,95]
- Invasomes (IVs): IVs are flexible and contain lipids, ethanol, and terpenes [23,96,97]
- Penetration enhancers containing vesicles (nPEVs): nPEVs are particles designed towards transdermal drug delivery, and have chemical penetration enhancers along with the basic components of LS, providing the ability to permeate biological barriers [23];

Yang et al. [84] optimized terbinafine TS formulations with different surfactants and incorporated them into a gel as a pharmaceutical vehicle. The optimized formulation containing Tween 80 was tested in vitro and in vivo. The in vitro permeation test using rat skin as a diffusion membrane demonstrated higher terbinafine release from the TS gel compared to a commercial cream, and the in vivo assay showed higher bioavailability of the TS gel compared to the same commercial product. Elsherif et al. [79] studied the encapsulation of terbinafine in SVs and assessed the drug permeation and retention in an ex vivo assay using human nails and observed that the terbinafine permeated and retained in the nails from SV formulations was 1.5-fold to 2-fold higher than that obtained with commercial terbinafine cream [79]

Bseiso et al. [23], prepared nPEVs with different permeation enhancers (N-acetyl-L-cysteine, thioglycolic acid, and thiourea) loaded with Sertaconazole for topical onychomycosis treatment and selected an optimal formulation using N-acetyl-L-cysteine (NAC) based on the deformability of the particles and high content of PE. The authors compared the optimal formulation with a commercial formulation and observed higher drug uptake by nail clippings and nail hydration potential from the nPEVs. [22]

Gupta et al. [62] evaluated a terbinafine-loaded invasomal gel formulation (TBF-INopt). The formulation contains linalool acting synergistically as a nail permeation enhancer. An in vitro release test demonstrated that the TBF-INopt gel presented controlled release of terbinafine (TBF) compared to the terbinafine suspension gel (control), and the nail permeation test carried out in Franz Diffusion Cells demonstrated that the vesicular system that carries terbinafine (TBF-INopt) penetrates 2.57 times more than the TBF suspension control.

Firooz et al. [63] performed an interventional pilot clinical study in patients with onychomycosis to test the efficacy and safety of a topical nanoliposomal gel carrying amphotericin B 0.4%. The study was based on monitoring 15 patients with onychomycosis undergoing treatment for a period of 12 to 36 weeks. The results of the study demonstrated that the tested formulation has minimal adverse effects and can be an alternative to conventional treatments.

Aiming to evaluate the differences in drug permeation across the nail barrier between LS and ES, Tuncay Tanriverdi [28] prepared LS and ES formulations containing caffeine as a model drug and performed permeation assays in Franz Diffusion Cells using human cadaver nails as diffusion membranes across 10 days and compared the formulations to the control groups that consisted of caffeine in aqueous and ethanolic solutions. It was observed that caffeine loaded into the VS had higher permeation coefficients than in solution, and that the ES promoted significant enhancement in permeation, even when compared to LS. The results were reinforced by nail topography images by Visioscan that demonstrated changes in nail surface upon treatment with VSs, with the ES system having the most notable effect, which is attributed by the author to the ethanol content in the formulations.

### 5.2. Microemulsions

Microemulsions (MEs) are nanometrical systems consisting of colloidal dispersions of hydrophobic and hydrophilic phases that present thermodynamic stability and have a transparent aspect. Although there is no agreement among authors, the particle size can range from 10 nm to 200 nm [92,98,99].

Barot et al. [20,21] performed formulation optimization studies using D-optimal Design and Box Behnken Design to encapsulate, respectively, terbinafine and itraconazole in MEs. Both papers observed better drug permeation and retention in skin [20,21] and skin and hooves [21] compared to a commercial formulation containing the respective drugs.

Thatai and Sapra [27] prepared MEs containing terbinafine HCl optimized by D-optimal design and investigated the effect of NAC and Urea alone and in different ratio combinations as permeation enhancers in hooves. The evaluation in PE showed that urea and NAC in association can present a synergistic effect, increasing the permeation of drugs across the hooves, which was confirmed by in vitro permeation tests.

### 5.3. Nanoemulsions

Nanoemulsions (NEs) are colloidal systems comprising two immiscible phases, one hydrophobic and one hydrophilic, in which one of the phases is dispersed in the other in the form of nanometric droplets stabilized by a surface-active agent. It is important to point out that NEs are kinetically stable but thermodynamically unstable, whilst the aforementioned MEs are thermodynamically stable systems [45,91,98].

Morgado et al. [17] tested the efficacy and safety of photodynamic therapy (PDT)-assisted delivery of Aluminium-Phthalocyanine Chloride (AlClPc) as a photosensitizer incorporated in NEs in a proof of concept clinical trial. The results indicate that the association of PDT with NE improved the rate of clinical cure compared to the conventional treatments with shorter treatment periods and an absence of adverse effects [17].

Mahtab et al. [82] demonstrated the incorporation of ketoconazole, a well-established drug used in the management of onychomycosis and other fungal infections, encapsulated in NE and subsequently evaluated the ex vivo permeation of NE containing ketoconazole in comparison to the drug in suspension in a goat hoof permeation model. The findings suggested that the incorporation of ketoconazole increased the permeation of the drug across the hoof when compared to the drug in suspension.

Nagalakshmi et al. [61] developed tolnaftate-loaded NEs incorporated into a topical nanoemulgel and evaluated drug release in vitro using the Franz Diffusion Cell method. The results demonstrated that the combination of two nanostructured systems, such as nanoemulsion and nanoemulgel, is an efficient alternative to the conventional treatment of onychomycosis, as it presents a sustained release of the drug.

### 5.4. Nanostructured Lipid Carriers and Solid Lipid Nanoparticles

Nanostructured lipid carriers (NLCs) are systems analogous to oil-in-water NEs in which the dispersed phase comprises a mixture of solid and liquid lipids. In many cases, the lipids and surfactants used in NLC formulations are already considered safe by regulatory institutions and used in the pharmaceutical and food industries. These systems have a relatively high drug loading capacity, being good candidates for the encapsulation of poorly soluble drugs [100,101].

In a review published in 2018, Gratieri, Krawczyk-Santos et al. [102] suggested NLCs and solid lipid nanoparticles (SLNs) as potential choices for the encapsulation of antifungal drugs intended for the treatment of onychomycosis. According to the authors, these systems present many advantages already observed upon cutaneous administration of such systems, which can be extrapolated to the nail administration due to alleged similarities between both administration routes. This suggests that both NLCs and SLNs are useful candidates in the development of new therapeutic alternatives for onychomycosis.

The first report of NLCs designed for topical treatment of onychomycosis by Rocha et al. [25] evaluated the potential of different PEs in the formulation using HEF24 h and SEM, leading to the choice of urea, and incorporated the antifungal Voriconazole into the formulation. In permeation studies using swine hooves, the NLC formulations with and without the PE presented higher retention of drug in the hooves compared to Voriconazole in solution. Both NLCs achieved concentrations higher than de MIC for dermatophytes.

A formulation of Ucúuba fat NLCs containing ketoconazole using a Quality by Design approach, employing Box Behnken Design, was described by Pereira et al. [76]. Two optimal formulations were selected and employed in drug release studies. These studies showed a dependency of the release profile related to the lipid-phase composition. The NLC formulations exhibited controlled release behavior compared to the immediate release profile of the drug in solution.

Abobakr et al. [73] prepared SLNs containing terbinafine and tested different PEs concerning their nail hydration potential and drug uptake by nail clippings. Among the tested PEs, thiourea was chosen for further investigation. The SLNs containing the PE presented higher drug uptake by nail clippings compared to a commercial formulation.

### 5.5. Polymeric Nanoparticles

Polymeric nanoparticles (PNPs), as in the case of NCs, are composed of polymers. The properties of the PNP are dependent on the choice of polymers for its composition. PNPs are matrixial systems, meaning that the drug loaded in these particles will be dissolved, entrapped, or attached to the polymeric matrix [88,103].

Chiu et al. [15] investigated the administration of a model system of PNPs associated with microporation of nail clippings as a potential drug delivery platform across the nail plate. To evaluate PNP permeation, a 7-day-long assay in Franz Diffusion Cells of fluorescently labelled PNPs in nail clippings was carried out, with stimulated Raman scattering and two-photon fluorescence imaging. It was possible to observe that the PNPs act as a reservoir of the drug to be released across several days and that microneedling created pores in the nail structure where the particles can release the drug and facilitate lateral diffusion to the diseased area.

Ullah et al. [64] and Kesharwani et al. [65] described the development of NPs using Chitosan, a naturally occurring polymer, containing terbinafine and itraconazole, with posterior incorporation of the NPs in gel vehicles, focusing on the topical treatment of onychomycosis with a sustained release profile of the drug molecules across the nail barrier.

### 5.6. Nanocapsules

Nanocapsules (NCs) are a “core-shell” type of structure built of a polymeric layer surrounding a hydrophobic or hydrophilic core with drug or bioactive molecules loaded within the polymeric layer or inside the core. The properties of the system, such as biocompatibility, tuning drug release, and biodegradability, can be modulated by the choice of polymer or polymer combination. In this sense, NCs can be very versatile [104,105,106]. A few studies were found in the literature that focused on developing nanocapsules for the topical treatment of nail disorders.

In the attempt to design a topical formulation aiming topical treatment of onychomycosis, Flores et al. [85] performed a comparative study between NCs and NEs containing tea tree oil as an antifungal agent. Both nanostructured systems presented better antifungal activity compared to a coarse emulsion. However, in release studies, NCs have been shown to sustain the release of chemical markers from tea tree oil for a longer period of time. Posterior studies from the same authors describe the development of NCs containing tioconazole aimed at topical treatment of onychomycosis, with the incorporation of NCs with and without cationic coating in a polymeric pharmaceutical vehicle. The authors compared the antifungal activity in an in vitro fungal infection model using human nail clippings and demonstrated that the NC formulations presented efficacy comparable to a commercial formulation, even though their tioconazole content was smaller [80]. Subsequently, the same formulation was investigated with respect to its ability to permeate the nail barrier, either associated or not associated with microporation of the nail. The authors observed that tioconazole from NCs demonstrated increased permeation compared to the commercial formulation. The nail poration approach yielded a significant effect when the formulation was applied weekly, but had no significant effect when the formulation was applied daily [16].

Morgado et al. [17] demonstrated the development and characterization of efinaconazole, an antifungal drug from the triazole subclass, loaded into poly(D,L-lactide-co-glycolide) nanocapsules [17], and subsequently evaluated the ex vivo permeation in bovine hoof membrane. A study of nail clippings was also carried out. The ENC formulation showed greater permeation compared to the reference formulation and microemulsion-gel loaded with efinaconazole (EFA). The nanocapsules showed that permeation is independent of EFA concentration, following a zero-order kinetic model.

### 5.7. Supramolecular Nanoparticles

Supramolecular nanoparticles (SMNPs) are nanostructured systems comprising self-assembled molecular building blocks stabilized by non-covalent intermolecular interactions, with a variety of possible applications, including in the biomedical field, such as drug delivery systems [107,108]. Wang et al. [78] described the encapsulation of ketoconazole in fluorescent SMNPs intended for intradermal deposition in areas adjacent to the nail via tattoo-mediated delivery as an alternative to the treatment of onychomycosis. The fluorescent marker incorporated in the system allowed the monitoring of the in vivo intradermal deposit concentration of ketoconazole in mouse skin. The correlation between fluorescence intensity and ketoconazole concentration demonstrated a prolonged controlled release profile.

### 5.8. Metal Nanoparticles

Metal nanoparticles (MNs) are popular inorganic nanoparticles. In addition, MNs are well known for having an intrinsic antimicrobial activity, which works through mechanisms different from the usual antimicrobial agents, thus overcoming the antimicrobial resistance problem and presenting an interesting alternative to the topical treatment of onychomycosis. Recently, efforts have been made towards the green synthesis of MNs using biodegradable materials such as plant extracts and microorganisms in biosynthetic processes as a more sustainable approach to obtaining MNs [109,110,111].

In this context, in an attempt to develop nanostructured systems for the topical treatment of onychomycosis, Dakhil, et al. [77] prepared AgNPs by fungal biosynthesis using Cladosporium cladosporioides [18] and biosynthesized AuNPs using plant extract of *Rosa indica* petals and loaded the particles with methylene blue, intending the system to be administered in association with photodynamic therapy to enhance the permeation of the system.

## 6. Pharmaceutical Vehicles

As addressed in previous sections, the treatment of nail disorders faces challenges, especially when related to the difficulty of obtaining therapeutic concentrations in the nail apparatus. In addition, topical treatment can be hindered by low time residence of conventional formulations like solutions, creams, and ointments. Notably, the removal of formulations due to routine activities can cause loss of drug content prior to permeation, imposing the need to focus on formulation strategies that favor the contact of the drug source to the nail surface [3,12].

In this context, several pharmaceutical vehicles are described in the literature as alternatives to the regular pharmaceutical formulations. For instance, drugs or delivery systems can be loaded in nail lacquers, gels, patches, and films that adhere to the nail surface. Such vehicles allow longer residence time of bioactive molecules, can increase nail hydration, and contain chemical permeation enhancers [3]. In addition, natural ingredients are safer alternatives to the use of solvents/chemicals that may induce changes in the microstructural characteristics of the nail plate [112,113].

Gels are one of the pharmaceutical vehicles described in the literature as alternatives for ungual administration of nanostructured systems. Hydrogels, in particular, have high water content and are expected to increase nail hydration, therefore loosening the keratin matrix of the nail plate, facilitating the passage of chemical entities through the structure [12,83]. Barot, et al. [20,21] demonstrated an in vitro increase in permeation and retention of drugs in the nail plate from MEs upon incorporation in hydrogels compared to MEs alone.

Nail lacquers are widely used in cosmetics both for aesthetic and protective purposes. Upon the application of a medicated nail lacquer, the solvent is expected to evaporate, leaving a film on the nail surface with a high concentration of the therapeutic agent. This creates a concentration gradient that would benefit the diffusion of molecules across the nail plate. The occlusive nature of the film prevents water loss and provides a hyperhydration on the most external regions of the nail plate, which is an important factor to enhance penetration of molecules across the nail [114,115,116]. Flores, et al. [80] and Flores, et al. [16] prepared tioconazole NCs and incorporated the nanostructures into a pullulan film-forming vehicle. When compared to an NC suspension, the NC in the film-forming vehicle exhibited a delayed release, but the overall amount of released tioconazole was higher. Shah and Jobanputra [81] prepared terbinafine LSs loaded in a nail lacquer using nitrocellulose as the film-forming agent and performed an in vitro drug permeation assay. The drug from the LS incorporated in the nail lacquer presented higher permeation rates than the drug dissolved in the nail lacquer, demonstrating the effect of the nanostructured system in enhancing ungual permeation.

## 7. Unanswered Questions

As discussed in the previous sections, all nanostructured systems tested in ungual permeation studies presented a permeation profile better than the control formulations, which varied across commercial formulations, e.g., lacquer, cream, solutions, and suspensions of the drug. Nevertheless, the comparison among different nanocarriers is impaired by many features. Firstly, there is a lack of standardization in the literature regarding the methodology of nail permeation/retention studies. The type of membrane used (cadaver nail, nail clippings, bovine hooves), diffusion cell, and temperatures used varied among the few studies that perform such assays, which are new to the literature compared to skin permeation studies. The majority of studies did not perform any evaluation of the ungual permeation. Some studies used skin modelling to suggest a potential application. Secondly, factors intrinsic to the formulation of each system, as well as the incorporation in different pharmaceutical vehicles, impair the comparison between different systems.

The lack of standardization of methodological approaches for the evaluation of transungual drug delivery goes beyond scientific research and represents a challenge to translational and regulatory frameworks. The need to discuss and harmonize an in vitro methodology able to assess drug permeation across the nail barrier and correlate the drug behavior in in vitro tests with in vivo performance is paramount. In this sense, a brief overview of the literature reveals that Franz Diffusion Cells using human nails, bovine, and porcine hooves were the most recurrent strategy for measuring drug permeation and retention in ungual tissues from different formulations, not limited to nanostructured systems.

Hydration has been suggested as one of the main mechanisms by which nail drug delivery is enhanced, as the increase in water content may cause loosening in the keratin network, favoring the diffusion of molecules across the nail matrix. But what might be the difference between the occlusive effect on nails of nanocarriers and conventional formulations? To propose mechanisms by which nanostructured formulations might improve permeation into the nail, a combination of analytical techniques and accurate selection of control formulations will be required. Even if nail hydration experiments assess the weight gain, the static feature of an ex vivo experiment may not reproduce the behavior in vivo. Formulations presenting similar viscosity and adherence should be compared in order to identify the effect of the nanocarrier.

Krawczyk-Santos, da Rocha [74] were the first to study protein dynamics by electron paramagnetic resonance (EPR). This technique provides evidence of any structure change, either by the hydration mechanism or by covalent or non-covalent bond alterations in nail modelling. This is a useful technique to evaluate all chemical permeation enhancers present in the nanocarriers and control formulation that weaken the physical and/or chemical bonds of the nail keratin matrix. Additionally, mercury intrusion porosimetry and image analysis by scanning electron microscopy are valuable tools in evaluating the impact of hydration in nails and bovine hoof membranes.

Similarly to the case with skin delivery, another proposed mechanism mentioned in some studies, although not yet proved, is the impact of low particle size and high surface area, with the presence of surfactants, on increasing the ability of nanosystems to be absorbed into the nail and move quickly through it.

## 8. Final Remarks

The pharmacological treatment of ungual disorders is challenging since this particular area presents a number of intrinsic features that make it difficult for therapeutic agents to reach the diseased area. Topical treatment, in particular, encounters the obstacle of the nail plate, which imposes a barrier to drug permeation. Many strategies have been employed aimed at increasing drug permeation across the nail. Among the many tools explored to achieve better drug permeation through the nail, the use of nanostructured systems has been shown to be a promising alternative. Recent studies demonstrate the potential of using nanotechnology both alone and in combination with other permeation enhancement techniques as a means to achieve satisfactory drug delivery to the nail. Among the different nanostructured systems covered in this review, vesicular systems stand out as the most investigated for promoting ungual permeation.

## Figures and Tables

**Table 1:** Methods employed to enhance drug permeation through the nail [7,10,47,54].

Mechanical	Physical	Chemical
Abrasion	Iontophoresis	Keratolytic enzymes
Nail avulsion	Etching	Solvents
	Laser therapy	Thiols and sulfites
	Electropulsation	Softening agents
	Ultrasonic therapy	Penetration enhancers
	Photodynamic therapy	Chemical etchants
	Microporation	
	Hydration	
	Occlusion	

**Table 2:** Publications found in the literature concerning the development of nanostructured systems designed for the local treatment of nail disorders.

Nanostructured System	API	In Vitro Permeation Assessment	Reference
NLC	Fluconazole and Riparin-B	In vitro permeation study using porcine hoof as diffusion membrane. It was not possible to measure permeated APIs, but authors observed retention of both drugs in hoof membrane.	[56]
SLN	Eficonazole and Fluconazole	NA	[57]
NE	Voriconazole and *Pinus silvestris* essential oil	NA	[58]
NLC	*Zataria multiflora* essential oil	NA	[59]
NP	Rose Bengal (dye)	NA	[60]
NE	Tolnaftate	NA	[61]
NC	Efinaconazole	An ex vivo permeation study using bovine hoof membranes demonstrated that the optimized nanocapsule formulation resulted in significantly greater API permeation compared to the reference formulation.	[22]
VS	Terbinafine	Linalool-incorporated vesicular systems presented an increase of approximately 2.5x in drug permeation using an in vitro permeation model using goat hooves as diffusion membranes. Additionally, it was observed that formulation containing vesicular systems associated with linalool as permeation enhancer allowed the observation of the drug in deeper regions of the hoof tissue.	[62]
VS	Amphotericin B	NA	[63]
NP	Terbinafine	In vitro permeation assay in Franz Diffusion Cells using human nail clipping as diffusion membrane. Authors compared NP to NP-loaded poloxamer gel containing terbinafine. No significant difference in ungual retention was observed between formulations; however, the presence of permeation enhancer was shown to increase drug permeation.	[64]
NP	Itraconazole and difluorated curcumin	In vitro permeation assay in Franz Diffusion Cells using bovine hoof as diffusion membrane. Authors compared the permeation of Itraconazole and Curcumin from NP-loaded gel and plain gel, denoting a sustained drug release profile from NP-loaded gel.	[65]
NP	Terbinafine	In vitro permeation assay in Franz Diffusion Cells using human cadaver nails as diffusion membrane. Authors compared the permeation of Terbinafine from NP-loaded gel and control gel. It was noticed that the incorporation of terbinafine in nanoparticles increased the permeation and provided a controlled-release drug profile.	[66]
VS	Eficonazole	NA	[67]
VS	Chorin e6	NA	[68]
NC	Ciclopirox	NA	[69]
VS	Itraconazole	NA	[70]
AgNP	NA	NA	[71]
Nanocomposites	Oxiconazole nitrate	NA	[72]
SLN	Terbinafine	NA	[73]
Nanomicelles	Voriconazole	In vitro permeation assay in Franz Diffusion Cells using bovine hoof as diffusion membrane. Authors compared VS, VS-loaded gel, and dispersion containing eficonazole. Incorporation in nanometric system was demonstrated to improve permeation across the barrier, with a slightly better performance when loaded into gel vehicle.	[74]
Pickering Emulsions	Tioconazole + tea tree oil	NA	[75]
NLC	Ketoconazole	NA	[76]
AgNP	NA	NA	[77]
ME	Ketoconazole	In vitro permeation assay in Franz Diffusion Cells using porcine skin as diffusion membrane. Authors compared ME-loaded gel containing ketoconazole to a commercial ketoconazole cream. No significant difference in skin retention was observed between formulations.	[19]
NC	Tioconazole	In vitro permeation assay in Franz Diffusion Cells using human nail clippings as diffusion membrane. Authors performed the experiment across 7 days, comparing a single administration and daily administration of Tioconazole containing NCs in porated and non-porated nail clippings. This study’s findings suggest that poration of nail had positive effect on tioconazole permeation and that a single administration of the delivery system to porated nail did not present a significant difference from daily administration.	[16]
VS	Caffeine (model drug)	In vitro permeation assay in Franz Diffusion Cells using human cadaver nails as diffusion membrane. Authors compared VSs (liposomes and ethosomes) containing caffeine (model drug) to caffeine dissolved in water and hydroalcoholic solution. Findings suggest that the incorporation of drug in vesicular systems increased drug permeation across the nail, being even more expressive in the presence of ethanol (ethosomes).	[28]
NP	Ketoconazole	NA	[78]
VS	Terbinafine	In vitro permeation assay in Franz Diffusion Cells using human cadaver nails as diffusion membrane. Authors compared VSs containing terbinafine to commercial formulation (Lamisil^®^ Cream). VSs presented permeation rates up to 2 times higher than the commercial formulation.	[79]
NC	Tioconazole	NA	[80]
NE	Aluminium- phthalocyanine chloride	NA	[17]
NLC	Voriconazole	In vitro permeation assay in Franz Diffusion Cells using animal hoof as diffusion membrane. Authors compared the permeation of unloaded voriconazole with that of NLC containing voriconazole with and without urea as a permeation enhancer. Incorporation of voriconazole in nanostructured system was shown to increase drug retention, but no difference was observed with the presence of urea.	[25]
VS	Terbinafine	NA	[81]
ME	Terbinafine	In vitro permeation assay in Franz Diffusion Cells using animal hoof as diffusion membrane. Authors compared terbinafine containing ME and ME incorporated into a gel vehicle, with and without the addition of chemical permeation enhancers. Findings show a correlation of use of permeation enhancers as a strategy to improve permeation, the incorporation of ME in gel vehicle with an increase in drug retention, and an increase in permeation with a decrease in particle size.	[27]
VS	Sertaconazole	NA	[23]
NE	Ketoconazole	In vitro permeation assay in Franz Diffusion Cells using goat hoof as diffusion membrane. Authors compared NE, NE incorporated into a gel vehicle, and a suspension containing ketoconazole. The cumulative amount of ketoconazole permeated from NE-gel was higher than from NE and suspension, which the authors attribute to the presence in the gel in the gel of thioglycolic acid effect as PE.	[82]
Au-NP	Au-NP and Methylene blue	NA	[18]
ZnO-NP	NA	NA	[83]
ME	Ciclopirox olamine	NA	[24]
VS	Terbinafine	In vitro permeation assay in Franz Diffusion Cells using human cadaver nails as diffusion membrane. Authors compared VSs containing terbinafine, prepared with different surfactants, loaded in polymeric films.	[29]
NP	NA	NA	[15]
VS	Terbinafine	In vitro permeation assay in Franz Diffusion Cells using human cadaver nails as diffusion membrane. Authors compared VSs containing terbinafine incorporated into Eudragit^®^ or Pululan^®^ films. Pululan films presented higher cumulative amounts of terbinafine detected in the diffusion membrane.	[84]
NE and NC	Tea tree oil	NA	[85]
VS	Terbinafine	In vitro permeation assay in Franz Diffusion Cells using human cadaver nails as diffusion membrane. Authors compared different types of VSs containing terbinafine. Findings suggest that VSs containing ethanol can increase drug permeation. Additionally, LS formulations incorporated into poloxamer gel presented better retention of drug compared to LS incorporated into chitosan gel.	[26]
ME	Itraconazole	In vitro permeation assay in Franz Diffusion Cells using stacked bovine hoof and human skin layers as diffusion membrane. Authors compared ME, ME incorporated into a gel vehicle, and commercial formulation containing itraconazole. ME and ME-gel showed better retention in the membranes, while commercial formulation was found to remain mostly between skin and hoof layers.	[21]
ME	Fluconazole	NA	[55]
ME	Terbinafine	In vitro permeation assay in Franz Diffusion Cells using human foot skin as diffusion membrane. Authors compared ME, ME incorporated into a gel vehicle, and commercial formulation containing terbinafine. ME presented higher permeation, and ME-gel showed better retention in the skin.	[20]

**Table 3:** Patents found in the literature concerning the development of nanostructured systems designed for the local treatment of nail disorders.

Patent Nº	Title	Year Priority	Purpose	Technology	Invention Summary
US10201571B2	Nanoparticle compositions and methods for treating onychomychosis.	2017	Onychomycosis treatment	Metallic nanoparticles	Silver metallic nanoparticles, containing or not containing a second metallic entity; in spherical format or “coral-shaped”; can contain or not contain a permeation enhancer; aiming at the treatment of ungual mycoses.
WO2011140126A2	Nail discoloration and fungus treatment.	2010	Onychomycosis and nail discoloration treatment	Silver nanoparticles	Silver nanoparticles/colloidal silver dispersed in gel vehicles (hydrogel, hydrogel moisture pads, hydrosol gels); application of nanosilver treatment followed by covering with hydrogel moist pad;
WO2015044669A1	Antifungal topical composition and methods of treatment.	2013	Topical treatment of mycosis	Polymeric nanoparticles	Nanoparticles formed by polymers derived from biguanidine capable of forming nanoparticles; containing an antifungal agent for topical administration in form of a cream, ointment, spray, or powder, and/or microneedle array, patch.
WO2017163091A1	Composition and methods of treatment.	2016	Treatment of nail and akin mycosis	Polymeric nanoparticles	Nanoparticles formed by polymers derived from biguanidine capable of forming nanoparticles; containing terbinafine or its respective salt and ethanol for topical administration.
WO2019002862A1	Nanoparticles formed of a polymer and terbinafine	2018	Treatment of onychomycosis and tinea pedis	Polymeric nanoparticles	Nanoparticles formed by polymers derived from biguanidine capable of forming nanoparticles; containing terbinafine or its respective salt and ethanol for topical administration by a spray device.
WO2020092884A2	Cross-linked supramolecular nanoparticles for controlled release of antifungal drugs and steroids–a new therapeutic approach for onychomycosis and keloid.	2018	Treatment of onychomycosis	Self-assembled supramolecular nanoparticles	Self-assembled supramolecular nanoparticles containing antifungal agent; containing reporter molecule (fluorescent reporter); designed for administration by penetration of epidermis layer (tattoo).

## Data Availability

The data supporting the review was addressed in Section 4 and will be made available upon reasonable request.

## Supplementary Materials

The following supporting information can be downloaded at: https://www.mdpi.com/article/10.3390/pharmaceutics17081060/s1. Supplementary Material S1—Query lines used in literature survey in each database. Supplementary Material S2—Composition and particle size of vesicular systems designed for ungual administration. Supplementary Material S3—Composition and particle size of microemulsions designed for ungual administration. Supplementary Material S4—Composition and particle size of nanoemulsions designed for ungual administration. Supplementary Material S5—Composition and particle size of NLC designed for ungual administration. Supplementary Material S6—Composition and particle size of NPs designed for ungual administration. Supplementary Material S7—Composition and particle size of nanocapsules designed for ungual administration. Supplementary Material S8—Preparation technique and particle size of mettalic nanoparticles designed for ungual administration.
