# Supplementary material for: Nanocarriers in Ungual Drug Delivery
Source: Pharmaceutics. 2025 Aug 15;17(8):1060. doi: 10.3390/pharmaceutics17081060 (PMC12389257; doi:10.3390/pharmaceutics17081060)
Supplement: Supplementary file 1 [file pharmaceutics-17-01060-s001.zip › pharmaceutics-3796612-supplementary.pdf]

Supplementary material 1 – Query lines used in literature survey in each database.

Database	Query line
Embase	(('ungual':ab,ti OR 'toenail*':ab,ti OR 'fingernail*':ab,ti OR 'onychomycosis':ab,ti OR 'mychosis nail':ab,ti OR 'nail mychosis':ab,ti OR 'nail fungal infection':ab,ti OR 'onychomychoses':ab,ti OR 'tinea unguium':ab,ti OR 'tinia unguium':ab,ti OR 'nail':ab,ti) AND 'psoriasis':ab,ti OR 'nail disease*':ab,ti OR 'nail disorder*':ab,ti OR 'onychopathy':ab,ti OR 'nail anomaly':ab,ti OR 'nail deformity'/exp OR 'nail deformity') AND ('nano*':ab,ti OR 'nanostructure*':ab,ti OR 'nanustructured drug delivery system*':ab,ti OR 'nanocapsule*':ab,ti OR 'nanoemulsion':ab,ti OR 'nano emulsion':ab,ti OR 'nano-emulsion':ab,ti OR 'nanogel':ab,ti OR 'solid lipid nanoparticle*':ab,ti OR 'nanostructured lipid carrier*':ab,ti OR 'liposome*':ab,ti OR 'phospholipid bilayer vesicle*':ab,ti)
Wed of Science	TI=(ungual OR toenail* OR fingernail* OR onychomycosis OR mychosis, nail OR nail mychosis OR nail fungal infection OR onychomychoses OR tinea unguium OR tinia unguium OR nail psoriasis OR nail disease* OR nail disorder* OR onychopathy OR nail anomaly OR nail deformity) AND TI=(nano* OR nanostructure* OR nanustructured drug delivery system* OR nanocapsule* OR nanoemulsion OR nano emulsion OR nano-emulsion OR nanogel OR solid lipid nanoparticle* OR nanostructured lipid carrier* OR liposome* OR phospholipid bilayer vesicle*)
Scopus	TITLE-ABS-KEY("ungual" OR "toenail*" OR "fingernail*" OR "onychomycosis" OR "mychosis, nail" OR "nail mychosis" OR "nail fungal infection" OR "onychomychoses" OR "tinea unguium" OR "tinia unguium" OR "nail psoriasis" OR "nail disease*" OR "nail disorder*" OR "onychopathy" OR "nail anomaly" OR "nail deformity") AND TITLE-ABS-KEY("nano*" OR "nanostructure*" OR "nanustructured drug delivery system*" OR "nanocapsule*" OR "nanoemulsion" OR "nano emulsion" OR "nano-emulsion" OR "nanogel" OR "solid lipid nanoparticle*" OR "nanostructured lipid carrier*" OR "liposome*" OR "phospholipid bilayer vesicle*")
Espacenet	(ta all "ungual" OR ta all "nail*" OR ta all "onychomycosis" OR ta all "nail psoriasis") AND (ta all "nano*" OR ta all "nanostructure*" OR ta all "nanosystem*")

Supplementary material 2 - Composition and particle size of vesicular systems designed for ungual administration.

Reference	Composition	Particle size (nm)
[1]	*Spanlastic nanovesicles*<br><br>Eficonazole<br><br>Ethanol<br><br>Tween 80<br><br>Deoxycholate<br><br>Span 60 or Span 65<br><br>Water	197
[2]	*Penetration enhancer containing vesicles*<br><br>Ce6<br><br>L-alpha-phosphatidilcholine<br><br>N-acetyl-L-cysteine<br><br>Chloroform<br><br>CTAB	264.29

	Phosphate buffer	
	Transcutol	
	Ethanol	
	Methanol	
	Water	
[3]	*Penetration enhancer containing vesicles*	193.55 – 252.5
	Itraconazole	
	N-acetyl-L-cysteine	
	Labrasol	
	Sterylamine	
	Chloroform	
	Metanol	
	Phosphate buffer	

[4]	*Liposomes*<br><br>Caffeine<br><br>Lipoid S 100 / Phospholipon 90 G<br><br>Cholesterol<br><br>Chloroform<br><br>PBS (pH: 7.4)	*Ethosomes*<br><br>Caffeine<br><br>Lipoid S 100<br><br>Cholesterol<br><br>Chloroform<br><br>Ethanol<br><br>Water	*Liposomes:*<br><br>545.3 and 610.2<br><br>*Ethosomes:*<br><br>349.5 and 337.9
[5]	*Spanlastic nanovesicles*<br><br>Terbinafine hydrochloride<br><br>Span ® 60<br><br>(in ethanol)<br><br>Span ® 65<br><br>(in acetone:chloroform 1:2(v/v))<br><br>Tween ® 80 / Sodium deoxycholate<br><br>Water		762.4 and 438.45 (optimized)

[6]	*Penetration enhancer containing vesicles*<br>Sertaconazole<br>Phosphatidylcholine<br>Transcutol P<br>Stearylamine<br>N-acetyl-L-cysteine / thioglycolic acid / thiourea<br>Chloroform:methanol 2:1 (v/v)<br>PBS (pH 7.4) / PBS (pH 7.4) + ethanol		66.5 (optimized)
[7,8]	*Liposomes*<br>Terbinafine hydrochloride<br>Lipoid S 100 / Phospholipon 90 G<br>Cholesterol<br>Chloroform<br>PBS (pH: 7,4)	*Ethosomes*<br>Terbinafine hydrochloride<br>Lipoid S 100 / Phospholipon 90 G<br>Ethanol<br>Water	*Liposomes*:<br>355.65 and 623.8<br>*Ethosomes*<br>188.5 and 225.3

[9]	*Transfersomes*<br><br>Terbinafine hydrochloride<br><br>Phospholipids<br><br>Sodium cholesteryl sulfate<br><br>Tween 80<br><br>Ethanol<br><br>PBS	69.6

Supplementary material 3 - Composition and particle size of microemulsions designed for ungual administration.

Reference	Composition	Particle size (nm)
[10]	Terbinafine<br>Oleic acid<br>Labrasol<br>Transcutol P<br>Water	18.14
[11]	Itraconazole<br>Oleic acid<br>Isopropyl myristate<br>Labrasol<br>Transcutol P<br>Water	48.2
[12]	Ciclopirox olamine<br>Capmul PG8<br>Cremophor EL	25.8

	Transcutol P<br><br>Water	
[13]	Terbinafine hydrochloride<br><br>Capmul MCM<br><br>Cremophor EL<br><br>Transcutol P<br><br>Water	22.15
[14]	Ketoconazole<br><br>Capryol<br><br>Nigella oil<br><br>Transcutol<br><br>Propylene glycol<br><br>Water	321.4

Supplementary material 4 - Composition and particle size of nanoemulsions designed for ungual administration.

Reference	Composition	Particle size (nm)
[15]	AlClPc (in ethanolic solution)<br>Cremophor ELP<br>Castor oil<br>PBS (pH=6,8)	30.4
[16]	Ketoconazole<br>Labrafac™<br>Lipophile WL 1349<br>Tween 80<br>PEG 400<br>Water	127 a 63.5
[17]	Tea Tree Oil<br>Span 80<br>Tween 80<br>Water	160

[18]	Ciclopirox olamine<br>Oleic acid<br>Tween 80<br>PEG 400<br>Water	25.51

Supplmentary material 5 - Composition and particle size of NLC designed for ungual administration.

Reference	Composition	Particle size (nm)
[19]	Voriconazole<br><br>Glyceryl behenate<br><br>Capric caprylic triglycerides<br><br>Polysorbate 80<br><br>Sorbitan trioleate<br><br>Cetylpyridinium chloride<br><br>Urea<br><br>Water	230
[20]	Ketoconazole<br><br>Ucuuba fat<br><br>TPGS 1000<br><br>Capryol™90<br><br>Water	33.6 and 74,6
[21]	Terbinafine	426.3 and 450.8 nm

Glyceryl Monostearate
Ethanol
Tween 80
Water

Supplementary material 6 - Composition and particle size of NPs designed for ungual administration.

Reference	Composition	Particle size (nm)
[22]	Terbinafine HCl<br><br>Lecithin<br><br>Tween 80<br><br>Chitosan<br><br>Ethanol<br><br>Acetic Acid<br><br>Water	229
[23]	Itraconazole<br><br>Difluorated Curcumin<br><br>DMSO<br><br>Sodium Tripolyphosphate<br><br>Sodium Hydroxide<br><br>Tween 20	207.2

	Chitosan<br>Acetic Acid<br>Water	
[24]	Terbinafine<br>Dichloromethane<br>Eudragit RSPO<br>Ethilcellulose<br>Polyvinyl Alcohol<br>Water	108.7
[25]	Nile Red<br>Octyl methoxycinnamate<br>5,10,15,20-tetrakis-<br>(4-aminophenyl)porphyrin<br>Acetone<br>Poly-ε-caprolactone	

	Tween 85
	Water

Supplementary material 7 - Composition and particle size of nanocapsules designed for ungual administration.

Reference	Composition	Particle size (nm)
[26]	Ciclopirox<br>Maisine<br>Acetone<br>Lipoid S75<br>poly(lactic-co-glycolic acid)<br>Span 60<br>Tween 20<br>Water	174.77
[27,28]	Tioconazole<br>Poly-ε-caprolactone<br>MCT<br>Acetone<br>Lipoid®S75 (in ethanolic solution)	155/157 (neutral)<br>162/166 (cationic, with chitosan coating)

	Polysorbate 80<br>Water	
[17]	Chitosan<br>Tea Tree Oil<br>Poly-ε-caprolactone<br>Acetone<br>Span 80<br>Tween 80<br>Water	220

Supplementary material 8 – Preparation technique and particle size of mettalic nanoparticles designed for ungual administration.

Reference	System	Preparation technique	Particle size (nm)
[29]	AgNPs	Biosynthesis using *Cladosporium cladosporioides*	5 – 50
[30]	ZnONPs	Biosynthesis using *Rosa indica*	50
[31]	AuNPs	Turkevich-Frens method	15

References

1. Almuqbil, R.M.; Sreeharsha, N.; Nair, A.B. Formulation-by-Design of Efinaconazole Spanlastic Nanovesicles for Transungual Delivery Using Statistical Risk Management and Multivariate Analytical Techniques. *Pharmaceutics* **2022**, *14*, doi:10.3390/pharmaceutics14071419.
2. Yasin, G.; Nasr, M.; Abdel Gaber, S.A.; Hüttenberger, D.; Fadel, M. Response Surface Methodological Approach for Optimization of Photodynamic Therapy of Onychomycosis Using Chlorin E6 Loaded Nail Penetration Enhancer Vesicles. *J Photochem Photobiol B* **2022**, *232*, doi:10.1016/j.jphotobiol.2022.112461.
3. Alqahtani, A.; Raut, B.; Khan, S.; Mohamed, J.M.M.; Fatease, A. Al; Alqahtani, T.; Alamri, A.; Ahmad, F.; Krishnaraju, V. The Unique Carboxymethyl Fenugreek Gum Gel Loaded Itraconazole Self-Emulsifying Nanovesicles for Topical Onychomycosis Treatment. *Polymers (Basel)* **2022**, *14*, doi:10.3390/polym14020325.
4. Tuncay Tanriverdi, S. Preparation and Characterization of Caffeine Loaded Liposome and Ethosome Formulations for Transungual Application. *Turk J Pharm Sci* **2018**, *15*, doi:10.4274/tjps.22931.
5. Elsherif, N.I.; Shamma, R.N.; Abdelbary, G. Terbinafine Hydrochloride Trans-Ungual Delivery via Nanovesicular Systems: In Vitro Characterization and Ex Vivo Evaluation. *AAPS PharmSciTech* **2017**, *18*, doi:10.1208/s12249-016-0528-9.
6. Bseiso, E.A.; Nasr, M.; Sammour, O.A.; Abd El Gawad, N.A. Novel Nail Penetration Enhancer Containing Vesicles “NPEVs” for Treatment of Onychomycosis. *Drug Deliv* **2016**, *23*, doi:10.3109/10717544.2015.1099059.
7. Tanriverdi, S.T.; Özer, Ö. Novel Topical Formulations of Terbinafine-HCl for Treatment of Onychomycosis. *European Journal of Pharmaceutical Sciences* **2013**, *48*, doi:10.1016/j.ejps.2012.12.014.
8. Tuncay Tanriverdi, S.; Hilmioƚlu Polat, S.; Yeşim Metin, D.; Kandiloƚlu, G.; Özer, Ö. Terbinafine Hydrochloride Loaded Liposome Film Formulation for Treatment of Onychomycosis: In Vitro and in Vivo Evaluation. *J Liposome Res* **2016**, *26*, doi:10.3109/08982104.2015.1067892.
9. Yang, Y.; Ou, R.; Guan, S.; Ye, X.; Hu, B.; Zhang, Y.; Lu, S.; Zhou, Y.; Yuan, Z.; Zhang, J.; et al. A Novel Drug Delivery Gel of Terbinafine Hydrochloride with High Penetration for External Use. *Drug Deliv* **2015**, *22*, doi:10.3109/10717544.2013.878856.
10. Barot, B.S.; Parejiya, P.B.; Patel, H.K.; Gohel, M.C.; Shelat, P.K. Microemulsion-Based Gel of Terbinafine for the Treatment of Onychomycosis: Optimization of Formulation Using D-Optimal Design. *AAPS PharmSciTech* **2012**, *13*, doi:10.1208/s12249-011-9742-7.

11. Barot, B.S.; Parejiya, P.B.; Patel, H.K.; Mehta, D.M.; Shelat, P.K. Microemulsion-Based Antifungal Gel Delivery to Nail for the Treatment of Onychomycosis: Formulation, Optimization, and Efficacy Studies. *Drug Deliv Transl Res* **2012**, *2*, doi:10.1007/s13346-012-0109-8.

12. Chouhan, P.; Saini, T.R. D-Optimal Design and Development of Microemulsion Based Transungual Drug Delivery Formulation of Ciclopirox Olamine for Treatment of Onychomycosis. *Indian J Pharm Sci* **2016**, *78*, doi:10.4172/pharmaceutical-sciences.1000145.

13. Thatai, P.; Sapra, B. Transungual Gel of Terbinafine Hydrochloride for the Management of Onychomycosis: Formulation, Optimization, and Evaluation. *AAPS PharmSciTech* **2017**, *18*, doi:10.1208/s12249-017-0711-7.

14. Amra, K.; Momin, M. Formulation Evaluation of Ketoconazole Microemulsion-Loaded Hydrogel with Nigella Oil as a Penetration Enhancer. *J Cosmet Dermatol* **2019**, *18*, doi:10.1111/jocd.12945.

15. Morgado, L.F.; Trávolo, A.R.F.; Muehlmann, L.A.; Narcizo, P.S.; Nunes, R.B.; Pereira, P.A.G.; Py-Daniel, K.R.; Jiang, C.S.; Gu, J.; Azevedo, R.B.; et al. Photodynamic Therapy Treatment of Onychomycosis with Aluminium-Phthalocyanine Chloride Nanoemulsions: A Proof of Concept Clinical Trial. *J Photochem Photobiol B* **2017**, *173*, doi:10.1016/j.jphotobiol.2017.06.010.

16. Mahtab, A.; Anwar, M.; Mallick, N.; Naz, Z.; Jain, G.K.; Ahmad, F.J. Transungual Delivery of Ketoconazole Nanoemulgel for the Effective Management of Onychomycosis. *AAPS PharmSciTech* **2016**, *17*, doi:10.1208/s12249-016-0488-0.

17. Flores, F.C.; de Lima, J.A.; Ribeiro, R.F.; Alves, S.H.; Rolim, C.M.B.; Beck, R.C.R.; da Silva, C.B. Antifungal Activity of Nanocapsule Suspensions Containing Tea Tree Oil on the Growth of Trichophyton Rubrum. *Mycopathologia* **2013**, *175*, doi:10.1007/s11046-013-9622-7.

18. Kumar, S.; Talegaonkar, S.; Negi, L.M.; Khan, Z.I. Design and Development of Ciclopirox Topical Nanoemulsion Gel for the Treatment of Subungual Onychomycosis. *Indian Journal of Pharmaceutical Education and Research* **2012**, *46*.

19. Rocha, K.A.D.; Krawczyk-Santos, A.P.; Andrade, L.M.; Souza, L.C. de; Marreto, R.N.; Gratieri, T.; Taveira, S.F. Voriconazole-Loaded Nanostructured Lipid Carriers (NLC) for Drug Delivery in Deeper Regions of the Nail Plate. *Int J Pharm* **2017**, *531*, doi:10.1016/j.ijpharm.2017.08.115.

20. Pereira, R.R.; Testi, M.; Rossi, F.; Silva Junior, J.O.C.; Ribeiro-Costa, R.M.; Bettini, R.; Santi, P.; Padula, C.; Sonvico, F. Ucuùba (Virola Surinamensis) Fat-Based Nanostructured Lipid Carriers for Nail Drug Delivery of Ketoconazole: Development and Optimization Using Box-Behnken Design. *Pharmaceutics* **2019**, *11*, doi:10.3390/pharmaceutics11060284.

21. Abobakr, F.E.; Fayez, S.M.; Elwazzan, V.S.; Sakran, W. Effect of Different Nail Penetration Enhancers in Solid Lipid Nanoparticles Containing Terbinafine Hydrochloride for Treatment of Onychomycosis. *AAPS PharmSciTech* **2021**, *22*, doi:10.1208/s12249-020-01893-9.
22. Ullah, K.H.; Rasheed, F.; Naz, I.; Ul Haq, N.; Fatima, H.; Kanwal, N.; Ur-Rehman, T. Chitosan Nanoparticles Loaded Poloxamer 407 Gel for Transungual Delivery of Terbinafine HCl. *Pharmaceutics* **2022**, *14*, doi:10.3390/pharmaceutics14112353.
23. Kesharwani, P.; Fatima, M.; Singh, V.; Sheikh, A.; Almalki, W.H.; Gajbhiye, V.; Sahebkar, A. Itraconazole and Difluorinated-Curcumin Containing Chitosan Nanoparticle Loaded Hydrogel for Amelioration of Onychomycosis. *Biomimetics* **2022**, *7*, doi:10.3390/biomimetics7040206.
24. Puri, V.; Froelich, A.; Shah, P.; Pringle, S.; Chen, K.; Michniak-Kohn, B. Quality by Design Guided Development of Polymeric Nanospheres of Terbinafine Hydrochloride for Topical Treatment of Onychomycosis Using a Nano-Gel Formulation. *Pharmaceutics* **2022**, *14*, doi:10.3390/pharmaceutics14102170.
25. Chiu, W.S.; Belsey, N.A.; Garrett, N.L.; Moger, J.; Price, G.J.; Delgado-Charro, M.B.; Guy, R.H. Drug Delivery into Microneedle-Porated Nails from Nanoparticle Reservoirs. *Journal of Controlled Release* **2015**, *220*, doi:10.1016/j.jconrel.2015.10.026.
26. Gaballah, E.Y.; Borg, T.M.; Mohamed, E.A. Hydroxypropyl Chitosan Nail Lacquer of Ciclopirox-PLGA Nanocapsules for Augmented in Vitro Nail Plate Absorption and Onychomycosis Treatment. *Drug Deliv* **2022**, *29*, doi:10.1080/10717544.2022.2144543.
27. Flores, F.C.; Rosso, R.S.; Cruz, L.; Beck, R.C.R.; Silva, C.B. An Innovative Polysaccharide Nanobased Nail Formulation for Improvement of Onychomycosis Treatment. *European Journal of Pharmaceutical Sciences* **2017**, *100*, doi:10.1016/j.ejps.2016.12.043.
28. Flores, F.C.; Chiu, W.S.; Beck, R.C.R.; da Silva, C.B.; Delgado-Charro, M.B. Enhancement of Tioconazole Ungual Delivery: Combining Nanocapsule Formulation and Nail Poration Approaches. *Int J Pharm* **2018**, *535*, doi:10.1016/j.ijpharm.2017.11.008.
29. Lafta, A.K.; Ajah, H.A.; Dakhil, O.A.A.; Ali AL-Wattar, W.M. Biosynthesis of Silver Nanoparticles Using Biomass of Cladosporium Cladosporioidesand Antifungalactivity against Pathogenic Fungi Causing Onychomycosis. *Plant Arch* **2019**, *19*.
30. Tiwari, N.; Pandit, R.; Gaikwad, S.; Gade, A.; Rai, M. Biosynthesis of Zinc Oxide Nanoparticles by Petals Extract of Rosa Indica L., Its Formulation as Nail Paint and Evaluation of Antifungal Activity against Fungi Causing Onychomycosis. *IET Nanobiotechnol* **2017**, *11*, doi:10.1049/iet-nbt.2016.0003.

31. Tawfik, A.A.; Noaman, I.; El-Elsayyad, H.; El-Mashad, N.; Soliman, M. A Study of the Treatment of Cutaneous Fungal Infection in Animal Model Using Photoactivated Composite of Methylene Blue and Gold Nanoparticle. *Photodiagnosis Photodyn Ther* **2016**, *15*, doi:10.1016/j.pdpdt.2016.05.010.
